# Renal Inflammation and Innate Immune Activation Underlie the Transition From Gentamicin-Induced Acute Kidney Injury to Renal Fibrosis

**DOI:** 10.3389/fphys.2021.606392

**Published:** 2021-07-07

**Authors:** Amanda Helen Albino, Fernanda Florencia Fregnan Zambom, Orestes Foresto-Neto, Karin Carneiro Oliveira, Victor Ferreira Ávila, Simone Costa Alarcon Arias, Antonio Carlos Seguro, Denise Maria Avancini Costa Malheiros, Niels Olsen Saraiva Camara, Clarice Kazue Fujihara, Roberto Zatz

**Affiliations:** ^1^Renal Division, Department of Clinical Medicine, Faculty of Medicine, University of São Paulo, São Paulo, Brazil; ^2^Laboratory of Transplantation Immunobiology, Institute of Biomedical Sciences, University of São Paulo, São Paulo, Brazil

**Keywords:** acute kidney injury, chronic kidney disease, gentamicin, innate immunity, NF-κB

## Abstract

Subjects recovering from acute kidney injury (AKI) are at risk of developing chronic kidney disease (CKD). The mechanisms underlying this transition are unclear and may involve sustained activation of renal innate immunity, with resulting renal inflammation and fibrosis. We investigated whether the NF-κB system and/or the NLRP3 inflammasome pathway remain activated after the resolution of AKI induced by gentamicin (GT) treatment, thus favoring the development of CKD. Male Munich-Wistar rats received daily subcutaneous injections of GT, 80 mg/kg, for 9 days. Control rats received vehicle only (NC). Rats were studied at 1, 30, and 180 days after GT treatment was ceased. On Day 1, glomerular ischemia (ISCH), tubular necrosis, albuminuria, creatinine retention, and tubular dysfunction were noted, in association with prominent renal infiltration by macrophages and myofibroblasts, along with increased renal abundance of TLR4, IL-6, and IL1β. Regression of functional and structural changes occurred on Day 30. However, the renal content of IL-1β was still elevated at this time, while the local renin-angiotensin system remained activated, and interstitial fibrosis became evident. On Day 180, recurring albuminuria and mild glomerulosclerosis were seen, along with ISCH and unabated interstitial fibrosis, whereas macrophage infiltration was still evident. GT-induced AKI activates innate immunity and promotes renal inflammation. Persistence of these abnormalities provides a plausible explanation for the transition of AKI to CKD observed in a growing number of patients.

## Introduction

Recent evidence indicates that, after recovering from widespread renal cellular necrosis and inflammation, a substantial fraction of patients who overcome acute kidney injury (AKI) develop an insidious process of renal interstitial collagen accumulation that eventuates in renal fibrosis (RF) and chronic kidney disease (CKD). The mechanisms underlying this transition from AKI to CKD are incompletely understood, and likely involve a complex interplay between renal infiltration by inflammatory cells and activation of innate immunity, adaptive immunity, and the local renal-angiotensin system (Hoste and Kellum, 2007; Chawla, 2011; Black et al., 2018; Sato and Yanagita, 2018).

Inflammatory phenomena are key to the development of kidney damage in both ischemic and nephrotoxic insults, the two most common causes of AKI. Early infiltration by inflammatory cells, along with increased expression of proinflammatory cytokines and adhesion molecules, has been reported in experimental AKI (Han and Bonventre, 2004; Kurts et al., 2013; Jang and Rabb, 2015; Li et al., 2018; Sato and Yanagita, 2018). Accordingly, AKI can be prevented by macrophage or lymphocyte depletion, as well as by cytokine inhibition (Han and Bonventre, 2004; Gonçalves et al., 2011; Zhang et al., 2013; Li et al., 2018). Inflammatory events also play a central role in the pathogenesis of RF and CKD, as shown with models as diverse as 5/6 renal ablation (Nx; Floege et al., 1992; Eddy, 1996; Fanelli et al., 2017; Foresto-Neto et al., 2018), diabetic nephropathy (Mezzano et al., 2004; Foresto-Neto et al., 2020), adriamycin nephropathy (Faustino et al., 2018), and unilateral ureteral obstruction (Esteban et al., 2004; Vilaysane et al., 2010), as well as in the AKI-CKD transition (Venkatachalam et al., 2015; Black et al., 2018; Yang, 2019).

In recent years, the role of innate immunity, particularly the NF-κB and NLRP3 inflammasome pathways, in the inflammatory process associated with AKI has become apparent (Iyer et al., 2009; Jang and Rabb, 2015; Li et al., 2018). Likewise, the prominent pathogenic role of innate immunity in CKD has been evidenced in a variety of experimental models (Jo et al., 2006; Fujihara et al., 2007; Iyer et al., 2009; Castoldi et al., 2012; Kim et al., 2013; Fanelli et al., 2017; Faustino et al., 2018; Zambom et al., 2019; Foresto-Neto et al., 2020).

In principle, activation of innate immunity by membrane debris and biomolecules released after renal ischemia/reperfusion (I/R) or toxic cell damage should be self-limited and cease once tubular regeneration is completed. However, if innate immunity activation persists, the resulting inflammation could lead to continuing renal damage. We hypothesized that, once set in motion in the context of AKI, the NF-κB system and/or the NLRP3 inflammasome pathway remain activated even after the acute event is resolved, thus sustaining an insidious process of renal inflammation that eventuates in RF, completing a complex transition to CKD.

To avoid the confounding effects of I/R-associated hypoxia (Nizet and Johnson, 2009; Mishra et al., 2015), thus focusing on the impact of cell injury on innate immunity, we utilized the model of nephrotoxic AKI by gentamicin (GT). Cytotoxicity caused by GT was shown to be associated with intense renal inflammation and NF-κB activation, along with residual renal accumulation of collagen 30 days after GT treatment (Nakajima et al., 1994; Geleilete et al., 2002; Volpini et al., 2004). However, whether GT-induced activation of renal innate immunity, inflammation, and fibrosis persists and progresses to CKD after longer periods of time has not been verified.

## Materials and Methods

### Experimental Protocol

All experimental procedures were approved by the Research Ethics Committee of the Faculty of Medicine of University of São Paulo (CEP-FMUSP, process no. 057/16). All experiments were performed in strict conformity with institutional guidelines and with international standards for manipulation and care of laboratory animals.

Sixty-three adult male Munich-Wistar rats, weighing 250–280 g, were obtained from a local facility at the Faculty of Medicine, University of São Paulo. The animals were maintained at 22 ± 1°C and 60 ± 5% relative air humidity, under an artificial 12:12 h light-dark cycle. Rats were fed regular chow containing 22% protein (Nuvital, Curitiba, Brazil) and *ad libitum* water.

The rats were divided into two groups: NC (*N* = 31), rats receiving subcutaneous injection of 0,15 M saline solution and GT (*N* = 32), rats receiving daily subcutaneous injections of GT (Gentatec-Chemitec, São Paulo, Brazil), 80 mg/kg/day, during 9 days. This protocol of GT administration standardized previously (Nakajima et al., 1994; Geleilete et al., 2002; Volpini et al., 2004) was calibrated to ensure that a toxic but nonlethal amount of GT reaches the kidneys, thus promoting tubular cell necrosis and AKI. Pretreatment body weights (267 ± 5 in NC and 265 ± 2 in GT) and albuminuria (1.6 ± 0.1 in NC and 1.5 ± 0.1 in GT) were similar between the two groups. NC and GT rats were studied 1 day (Day 1, 10 NC and 10 GT rats), 30 days (Day 30, 10 NC and 10 GT rats), and 180 days (Day 180, 11 NC and 12 GT rats) after these 9-day treatments were ceased. At each time point, body weight (BW) and systolic blood pressure were determined using an automated optoelectronic device (BP 2000 Blood Pressure Analysis System, Visitech Systems, EUA). All rats were preconditioned to remain calm during the procedure. In addition, animals were kept for 24 h in metabolic cages for urine collection for measurement of albumin and creatinine excretion.

On Days 1, 30, and 180, rats were anesthetized with ketamine (50 mg/kg im.) and xylazine (10 mg/kg im.). Blood samples were taken from the abdominal aorta for measurement of serum creatinine and plasma sodium and potassium concentrations. The kidneys were retrogradely perfused *in situ* through the abdominal aorta with cold saline to remove blood from renal vessels. The right kidney was excised, instantly frozen in liquid nitrogen, and stored at −80°C for subsequent analysis. The left kidney was retrogradely perfused *in situ* with Duboscq-Brazil solution for fixation. The renal tissue was then weighed, cut in two mid-coronal slices, post-fixed in buffered 10% formaldehyde solution, and embedded in paraffin, using standard sequential techniques, for histomorphometric and immunohistochemical analysis, performed in 4-μm-thick sections. Rats were killed instantly by the kidney perfusion-fixation procedure.

### Biochemical and Enzymatic Analysis

Urinary albumin was determined by radial immunodiffusion (Mancini et al., 1965) using a polyclonal rabbit anti-albumin antibody (#0855715, MP Biomedicals LLC, United States). Serum and urine creatinine concentrations were measured by a colorimetric assay kit (Labtest Diagnostic, Sao Paulo, Brazil). The urine albumin/creatinine ratio was expressed in mg/mg.

### Histomorphometric Analysis

The morphometric evaluations were performed in a blinded manner by a single observer. The extent of glomerular injury was estimated by determining the percentage of glomeruli with either ischemic (ISCH) or sclerotic lesions (GS) in sections stained by the Periodic acid-Schiff reaction (Fujihara et al., 1994). Glomerular ischemia was defined as a collapse of the glomerular tuft, with decrease in the tuft volume, closure of the capillary loops, wrinkling of the basement membrane, and enlargement of the Bowman’s space. GS was characterized by deposition of hyaline material in a segment of the glomerular tuft, with consequent occlusion of capillary loops.

### Immunohistochemical Analysis

Renal slices were mounted on glass slides coated with 6% silane. The following primary antibodies were employed as: monoclonal mouse anti-ED-1 (#MCA341R, Serotec, Oxford, United Kingdom) for macrophages, polyclonal rabbit anti-Mannose receptor (CD206, #Ab64693, Abcam, Cambridge, United Kingdom) for M2 macrophages (anti-inflammatory phenotype), monoclonal mouse anti-α-smooth muscle actin (α-SMA; #A2547, Sigma-Aldrich, Saint Louis, MO), polyclonal rabbit anti-collagen type 1 (#34710, Abcam, Cambridge, United Kingdom), polyclonal rabbit anti-fibronectin (#Ab2413, Abcam, Cambridge, United Kingdom), and polyclonal rabbit anti-angiotensin II (AngII; #T4007, Peninsula Laboratories, San Carlos, CA) for AngII-positive cells. The immunohistochemical techniques used in this study were described in detail in the previous studies (Arias et al., 2013; Foresto-Neto et al., 2018; Zambom et al., 2019). The interstitial density of macrophages and AngII-positive cells was evaluated in a blinded manner at ×400 magnification. For each section, 25 microscopic fields (corresponding to a total area of 0.08 mm^2^) were examined. Results were expressed in cells/mm^2^. The percentage of cortical interstitial area occupied by collagen-1 and fibronectin was estimated by a point-counting technique (Jepsen and Mortensen, 1979).

### Total Protein Extraction

Kidney proteins were extracted using lysis buffer (#89900, Thermo Scientific, Rockford, IL) with protease and phosphatase inhibitor (Roche, Mannheim, Germany). Protein concentration was determined with the bicinchoninic acid method, using homogenate aliquots containing 100 μg of protein.

### Western Blot Assays

A 100 μg aliquot of renal homogenate was mixed in 2 × Laemmli buffer and denatured at 96°C for 5 min. For the specific nuclear fraction analysis, the pre-prepared samples were not denatured. Protein separation was performed by sodium dodecyl sulfate-polyacrylamide gel electrophoresis. For each blot, samples from two rats per group were loaded so that the intensity of each band (factored by its respective housekeeping band) could always be reliably compared among groups. Seven rats per group per time point were analyzed in this way. The separated proteins were transferred to a nitrocellulose membrane, which was incubated with 5% non-fat milk or 5% BSA in tris-buffered saline for 2 h at room temperature to block nonspecific binding. The membrane was then incubated overnight at 4°C with primary antibodies for: monoclonal mouse anti-β-actin, 1:5,000 (#A2228, Sigma-Aldrich, Saint Louis, MO); polyclonal rabbit anti-TLR4 (#Sc30002, Santa Cruz Biotechnology, Dallas, TX); monoclonal mouse anti-caspase-1 (casp-1), 1:1,000 (#Sc56036, Santa Cruz Biotechnology, Dallas, TX); monoclonal mouse anti-interleukin 6 (IL-6), 1:1,000 (#Ab9324, Abcam, Cambridge, United Kingdom); and monoclonal mouse anti-αSMA, 1:1,000 (#A2547, Sigma-Aldrich, Saint Louis, MO). After rinsing with Tris-buffered saline Tween 20 buffer, membranes were incubated with secondary antibodies labeled with HRP. Immunostained bands were detected using a chemiluminescence kit (Thermo Scientific, Rockford, United States) and were further analyzed by densitometry with a gel documentation system and the Uvisoft-Uviband Max software (Uvitec Cambridge, Cambridge, United Kingdom).

### ELISA Analysis

The renal contents of KIM-1 and IL-1β were determined using a commercial ELISA kit (R&D Systems, Minneapolis, MN). The analyses were performed following rigorously the manufacturer’s instructions.

### Statistical Analysis

Statistical differences among groups were assessed by one-way ANOVA, with pairwise post-test comparisons according to the Tukey’s method (Wallenstein et al., 1980). The differences were considered significant at *p* < 0.05. Results were expressed as means ± *SE*. All calculations were performed using the GraphPad Prism 6.01 software.

## Results

**Figure 1:**
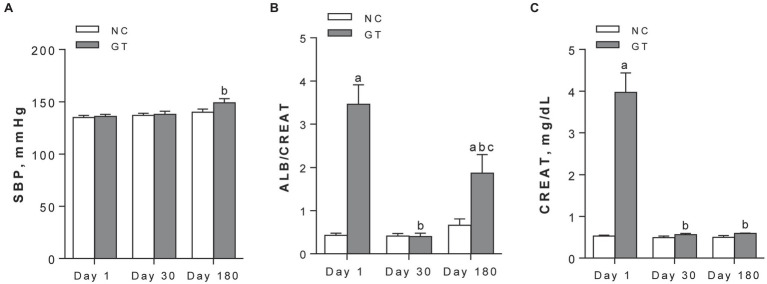
Systolic blood pressure **(A)**, urine albumin/creatinine ratios, in mg/mg **(B)**, and serum creatinine **(C)** assessed on Days 1, 30, and 180 after GT treatment in groups NC and GT. Results expressed as means ± *SE*. ^a^*p* < 0.05 vs. NC; ^b^*p* < 0.05 vs. Day 1; ^c^*p* < 0.05 vs. Day 30.

Although body weight was not significantly changed by GT on Day 1 (258 ± 3 g vs. 262 ± 8 g in NC, *p* > 0.05), growth was limited on Day 30 (281 ± 6 g vs. 298 ± 10 g, respectively, *p* < 0.05). On Day 180, body growth was similar between groups. Whereas group GT remained normotensive on Day 1 and Day 30, BP was modestly but significantly (*p* < 0.05) elevated on Day 180 compared to Day 1 (Figure 1A). GT rats developed a marked increase in the albumin excretion rate on Day 1 (21.4 ± 3.0 mg/24 h vs. 3.5 ± 0.6 in control, *p* < 0.05). Albuminuria regressed on Day 30 (2.9 ± 0.6 mg/24 h vs. 2.3 ± 0.4 in control, *p* > 0.05), but returned on Day 180 (25.0 ± 5.5 mg/24 h vs. 7.5 ± 2.1 in control, *p* < 0.05). Parallel changes were noted in the albumin/creatinine ratio (Figure 1B). Serum creatinine was prominently elevated on Day 1, returned to control on Day 30, and remained at normal levels on Day 180 (Figure 1C).

**Figure 2:**
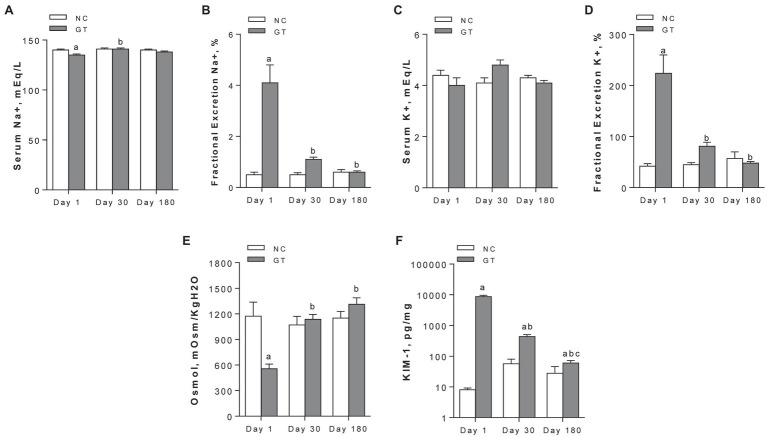
Serum concentration **(A)** and fractional excretion **(B)** of sodium; serum concentration **(C)** and fractional excretion **(D)** of potassium; and urinary osmolality **(E)** and renal content of KIM-1 **(F)**. Results expressed as means ± *SE*. ^a^*p* < 0.05 vs. NC; ^b^*p* < 0.05 vs. Day 1; ^c^*p* < 0.05 vs. Day 30.

On Day 1, GT rats exhibited a slight but significant decrease in serum sodium levels, while a marked increase in the fractional excretion of sodium (FE_Na+_) and potassium (FE_K+_) was observed. Both parameters returned to control on Day 30 and remained at these levels on Day 180 (Figures 2A–D). Urine osmolality was markedly reduced in GT rats on Day 1, returning to control levels from Day 30 on Figure 2E. The renal content of KIM-1 was strikingly increased on Day 1 (*p* < 0.0001), and declined progressively on Days 30 and 180, though remaining slightly but significantly increased above control levels (Figure 2F).

**Figure 3:**
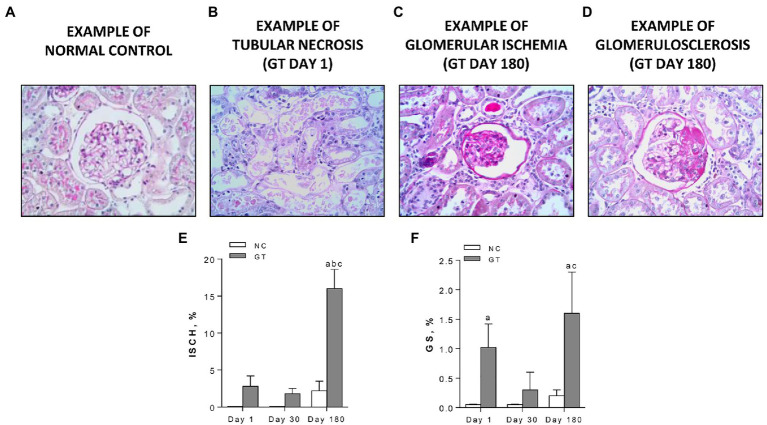
Representative microphotographs of renal lesions (PAS-stained, 400×) **(A)** normal glomerulus; **(B)** widespread acute tubular necrosis, present on Day 1 only, with tubular profiles nearly completely devoid of cells and filled by necrotic material; **(C)** a representative ischemic (ISCH) glomerulus, showing tuft collapse, wrinkled basement membrane, and closure of capillary loops, on Day 180; **(D)** a representative glomerulus with a segmental sclerotic lesion (GS), with occlusion of capillary loops by a hyaline material, on Day 180; and **(E,F)** quantitative analysis of ISCH and GS, respectively, in groups NC and GT. Results expressed as means ± *SE*. ^a^*p* < 0.05 vs. NC, ^b^*p* < 0.05 vs. Day 1, and ^c^*p* < 0.05 vs. Day 30.

On Day 1, GT-treated rats exhibited widespread acute tubular necrosis (ATN), with cell lysis and cellular debris in most tubular lumina. Glomerulosclerosis (GS), with segmental areas of mesangial expansion and capillary loop occlusion, as well as glomerular ischemia (ISCH), with collapse of the entire tuft and closure of capillary loops, were also observed. Representative microphotographs of ATN, ISCH, and GS, along with the frequency of ISCH and GS on Days 1, 30, and 180, are shown in Figure 3. Widespread ATN was observed on Day 1, but complete tubular cell regeneration was evident on Days 30 and 180. Percent ISCH was slightly and not significantly increased on Days 1 and 30 but was clearly elevated on Day 180 (*p* < 0.001). The frequency of GS was markedly increased on Day 1, regressed on Day 30, but returned to abnormally high levels on Day 180.

**Figure 4:**
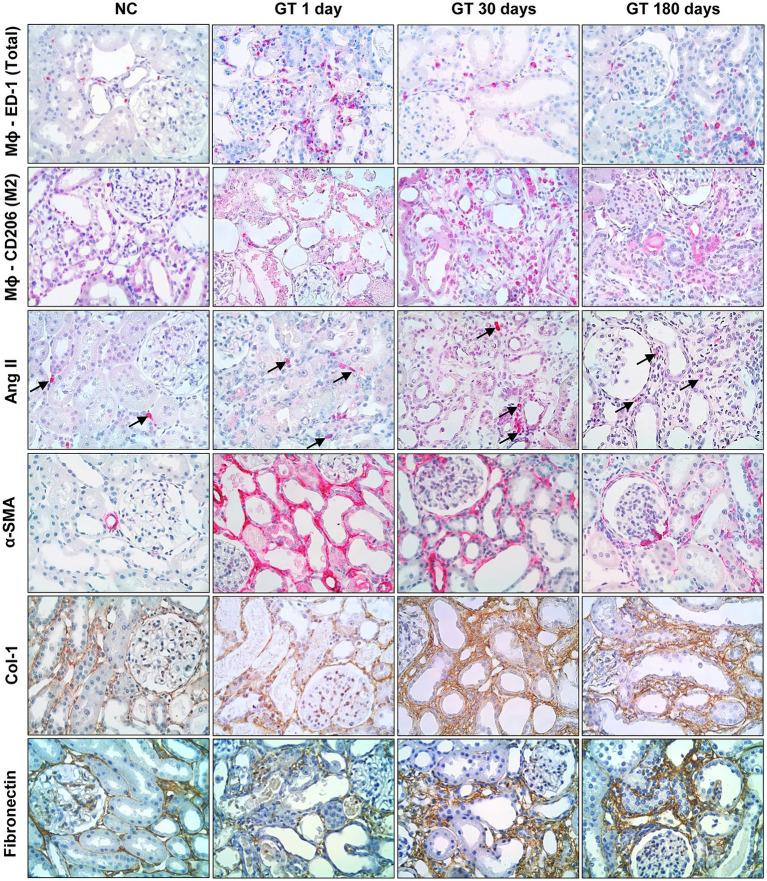
Representative microphotographs (in immunohistochemically stained sections, 400×) of renal interstitial infiltration by total macrophages (ED-1-positive cells); M2 macrophages (CD206-positive cells); AngII-positive cells; α-SMA-positive cells; and renal interstitial deposition of Collagen-1 and Fibronectin in groups NC and GT on Days 1, 30, and 180.

**Figure 5:**
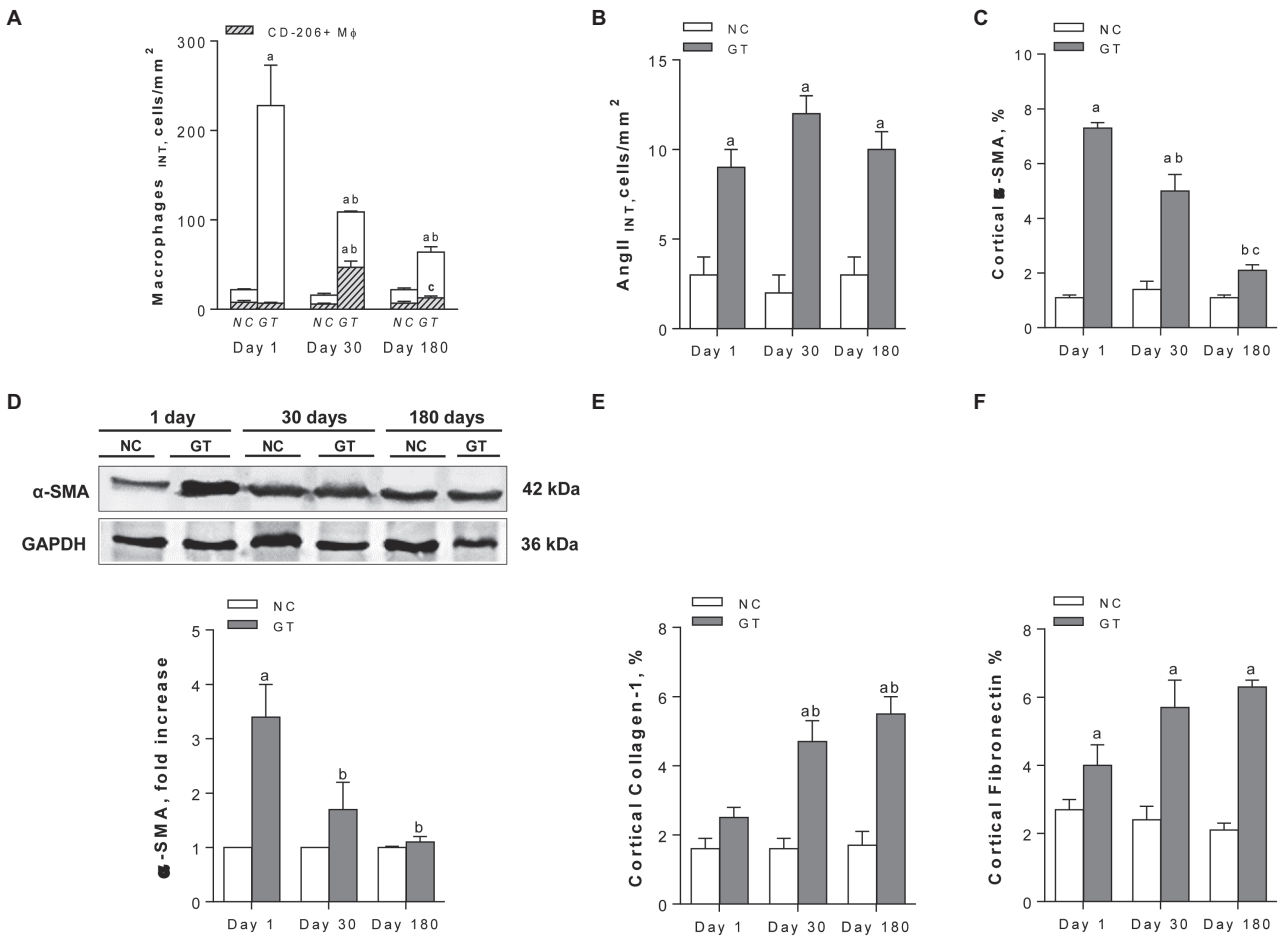
Quantitative analysis. **(A)** interstitial macrophages, with the CD206-positive fraction represented by the hatched areas; **(B)** interstitial AngII-positive cells; **(C)** percent cortical α-SMA-positive area (by immunohistochemistry); **(D)** renal abundance of α-SMA (by Western blot); and **(E,F)** percent Collagen-1- and Fibronectin-positive cortical areas, respectively (by immunohistochemistry), in groups NC and GT on Days 1, 30, and 180. Western blot analysis included seven animals per group. Results expressed as means ± *SE*. ^a^*p* < 0.05 vs. NC, ^b^*p* < 0.05 vs. Day 1, and ^c^*p* < 0.05 vs. Day 30.

Representative microphotographs of renal tissue stained by immunohistochemistry, focusing on the expression of inflammatory components along the study, are shown in Figure 4, whereas the corresponding quantitative analyses are given in Figure 5. On Day 1, GT rats exhibited intense renal interstitial infiltration by macrophages. Of note, M2-type anti-inflammatory cells constituted only a small fraction of the infiltrating macrophages. AngII-positive cells and myofibroblasts were also conspicuously present at this phase. On Day 30, the intensity of macrophage infiltration was attenuated, but the anti-inflammatory M2 phenotype now represented nearly half of its composition. The presence of myofibroblasts was also strongly reduced at this time, while that of AngII-positive cells remained unabated. On Day 180, low-grade renal interstitial macrophage infiltration was still observed, whereas myofibroblasts had nearly returned to control levels. By contrast, the number of AngII-positive cells remained at the same elevated levels as observed on Days 1 and 30 (*p* < 0.0001 vs. NC).

Representative microphotographs showing renal tissue stained by immunohistochemistry for collagen-1 and fibronectin are shown in Figure 4. Renal deposition of both molecules exhibited a progressive increase along the study, becoming significantly elevated compared to NC, and indicating that an insidious process of fibrosis took place in the renal tissue (Figure 5).

**Figure 6:**
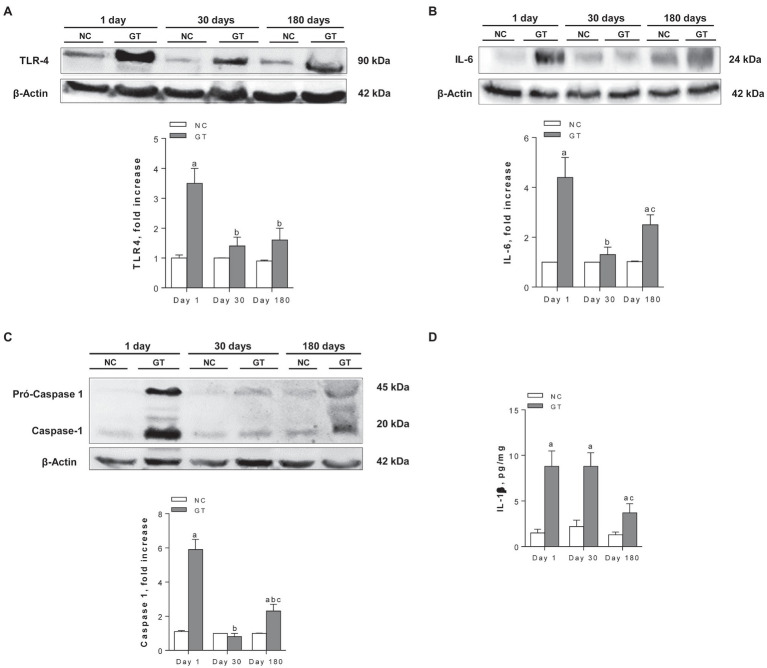
Representative Western blots and quantitative assessment of the abundance of Toll-like receptor 4 **(A)**, Interleukin-6 **(B)**, Caspase-1 **(C)**, and renal content of IL-1β by ELISA **(D)** in groups NC and GT on Days 1, 30, and 180. Western blot analysis included seven animals per group. Results expressed as means ± *SE*. ^a^*p* < 0.05 vs. NC, ^b^*p* < 0.05 vs. Day 1, and ^c^*p* < 0.05 vs. Day 30.

The renal contents of TLR4, IL-6, the active form of caspase-1, and IL-1β were all increased in GT rats on Day 1 (Figure 6). All these parameters returned to normal on Day 30, except for IL-1β. On Day 180, IL-1β remained significantly increased compared to controls, whereas IL-6 and caspase-1 were again elevated.

## Discussion

As shown previously (Nakajima et al., 1994; Geleilete et al., 2002; Volpini et al., 2004), GT rats developed nephrotoxic AKI, with limited body growth, marked creatinine retention, and tubular damage, characterized by clear histologic evidence of cellular necrosis, along with marked increase in the renal content of KIM-1. These tubular structural changes were accompanied by severe functional limitation, with a striking increase in the fractional excretion of sodium and potassium, as well as a drastic fall in urine osmolality. In addition, a marked increase in the urine albumin/creatinine ratio and low-grade sclerosing tuft lesions indicated that the glomeruli were also damaged.

An intense macrophage infiltration was shown on Day 1. Only few of these cells were CD206-positive, indicating that the M2 subtype did not play a role at this acute phase, and that macrophages followed a predominantly proinflammatory behavior, as observed in the previous studies of this and other AKI models (Geleilete et al., 2002; Jo et al., 2006). Cells staining positively for α-SMA, presumably myofibroblasts, were also detected at this stage, in agreement with the previous studies of either the GT (Geleilete et al., 2002) or the I/R model (Forbes et al., 2000). Infiltration by cells staining positively for Ang II was also observed, indicating local activation of the renin-angiotensin system (RAS), not previously shown in association with GT nephrotoxicity. This observation is consistent with the observed overexpression of α-SMA, and with the report of a beneficial effect of losartan in experimental GT nephrotoxicity (Heeba, 2011). Together, these findings reinforce the notion that ATN, whether resulting from I/R or toxicity, is associated with exuberant renal inflammation (Forbes et al., 2000; Geleilete et al., 2002; Iyer et al., 2009). Of note, renal fibronectin accumulation was also observed, indicating that incipient renal fibrosis was already present at this early stage.

The renal inflammation observed on Day 1 in rats that received GT was associated with a marked increase in the abundance of IL-6, one of the main NF-κB targets. This finding is consistent with that of Volpini et al. (2004), who reported activation of the NF-κB system 5 days after GT administration. In addition, treatment with GT led to an increase in the renal abundance of TLR4, caspase-1, and IL-1β, suggesting that the NLRP3 inflammasome pathway was also activated. Since what was measured was total IL-1β, it cannot be established with certainty whether the renal content of its active form was also increased. However, as the renal abundance of caspase-1 was also augmented, it is more than likely that this was indeed the case.

Taken together, these results indicate that a strong activation of innate renal immunity occurred on Day 1, encompassing both the NF-κB and NLRP3 pathways. The mechanisms that triggered this process have not been determined. Cell remains, such as membrane debris and DNA fragments, can function as danger-associated molecular patterns (DAMPs), binding to TLRs and activating both the NF-κB and NLRP3 inflammatory pathways (Anders et al., 2004; Iyer et al., 2009; Martinon et al., 2009; Lorenz et al., 2014). In addition, the NF-κB system may have been activated by AngII and/or by the presence of oxidative stress, as demonstrated in other models (Ruiz-Ortega et al., 2000; Mezzano et al., 2004; Ji et al., 2009; Heeba, 2011). Irrespective of the triggering factors, this process helps to explain the development of the intense renal inflammatory process that involved the kidneys during the acute phase of GT toxicity. It is noteworthy that all these changes took place only 1 day after the GT injections, suggesting that they constitute a fast response to cell damage/destruction.

The structural changes observed on Day 1 had completely regressed 30 days after GT administration. The glomerular sclerosing lesions nearly disappeared, indicating that they had reflected readily reversible processes, such as mesangial matrix deposition, with no permanent structural damage. The associated functional changes also regressed, with normalization of plasma creatinine and albuminuria. There was also full recovery from tubular necrosis, with cell regeneration and return of the renal KIM-1 content to normal values. Tubular function was also restored, with normalization of fractional sodium and potassium excretion, in addition to full recovery of urine concentrating ability.

Renal inflammation also regressed on Day 30. Macrophage infiltration lost intensity, while subtype 2 (CD-206-positive) anti-inflammatory cells now represented 45% of the total cell count, compared to 6% on Day 1. Likewise, myofibroblast infiltration declined to values no longer significantly different from those observed in NC. Together, these findings seemed to indicate that, 1 month after the GT insult, the intense inflammatory process observed on Day 1 had ceased and was on the way to complete resolution. However, this regression was only partial. The density of Ang II-positive cells remained as high as observed on Day 1. In addition, collagen type-1 deposition, which was not significantly increased on Day 1, was now clearly augmented, accompanied by a similar increase in fibronectin accumulation, each taking up a much larger fraction of the renal cortex than in control rats. Innate immunity also remained activated as: although the renal contents of caspase-1 and TLR-4 were normalized, IL-1β and the p65 moiety remained at levels similar to those observed on Day 1. Thus, a silent inflammatory process, associated with, and likely fueled by, an equally quiescent activation of innate immunity, was operative at this time point.

In the present study, rats acutely exposed to the nephrotoxic effects of GT were followed for an unprecedented 180 days after treatment. At the end of this period, only residual signs of tubular injury remained, as indicated by a decrease of urinary KIM-1 to levels only slightly above control, while the fractional excretion of sodium and potassium, as well as urinary osmolality, stayed at control levels. However, several signs of an insidious CKD were now apparent. Although serum creatinine remained close to normal, glomerular sclerosing lesions, which had regressed on Day 30, now reappeared; a considerable fraction of the glomeruli exhibited changes characteristic of ischemia; and albuminuria returned with the same intensity observed on Day 1. These findings indicate that, despite the apparent regression observed on Day 30, the glomerular injury initiated by GT administration remained active.

The renal inflammation observed on Day 1 also appeared to have completely subsided on Day 180, with macrophages (both total and CD206-positive) and myofibroblasts down to normal values. However, other findings indicated that low-grade renal inflammation persisted. Cortical infiltration by AngII-positive cells remained unabated, indicating continued RAS activation. Moreover, renal accumulation of collagen-1 and fibronectin was still evident, indicating persistence of renal fibrosis. As to innate immunity, the renal content of IL-1β was still abnormally high, albeit at more modest levels than initially observed, while the abundance of caspase-1, which had returned to normal on Day 30, was once again heightened. On the other hand, the abundance of IL-6 was still significantly elevated compared to NC values, consistent with persistent activation of the NF-κB system.

The development of insidious renal inflammation following resolution of an acute insult has been reported in our laboratory and elsewhere utilizing differing experimental models of CKD. Rodríguez-Iturbe et al. showed that rats undergoing temporary treatment with L-NAME developed permanent renal inflammation and salt-sensitive arterial hypertension (Rodríguez-Iturbe et al., 2012; Franco et al., 2013). In our study, renal inflammation, along with increased local AngII production, may help to explain why hypertension developed after AKI had subsided. It must be noted that angiotensin II produced outside the afferent arteriole acts much as a proinflammatory cytokine, rather than promoting hypertension by a systemic effect. This dichotomy helps to explain the dissociation between blood pressure and the amount of cells staining positively for angiotensin II, observed in this and in the previous studies (Mattar et al., 2007; Franco et al., 2013).

Slowly progressive nephropathy after acute injury was also shown by Anderson et al. (1988), who described the development of progressive glomerulosclerosis after treatment with puromycin aminonucleoside that led to short-lived massive proteinuria. Likewise, Fujihara et al. (2006) and Mattar et al. (2007) showed that rats subjected for a short period to NO inhibition by L-NAME developed progressive glomerulosclerosis associated with renal interstitial fibrosis. The reasons for these transitions have not been elucidated. In the present study, CKD could result from a marked reduction in the number of nephrons, leading to overload of the remaining glomeruli and intracapillary hypertension, as previously demonstrated for other CKD models (Anderson et al., 1988; Fujihara et al., 2000, 2007). However, this explanation seems unlikely, since creatinine retention was not observed on Days 30 and 180. Moreover, the intensity of glomerular damage was only moderate, less than 2% of glomeruli showing sclerosing lesions. An attractive possibility is the lasting activation of the local RAS, previously demonstrated in other CKD models (Fujihara et al., 2006; Mattar et al., 2007), which could help explain both glomerular and tubulointerstitial injury. The mechanism of continued RAS activation after recovery from GT-induced AKI is uncertain. It is noteworthy that evidence of equally persistent NF-κB and NLRP3/IL-1β activation was also observed in GT rats. AngII can activate the NF-κB system by degrading the I-κB inhibitory factor (Ruiz-Ortega et al., 2000; Douillette et al., 2006). Conversely, activation of the NF-κB system stimulates the expression of the AT1 receptor (Klahr and Morrissey, 2000; Luo et al., 2015). Similar interactions have been shown between the NF-κB and the NLRP3/IL-1β systems (Solt et al., 2007; Verstrepen et al., 2008; Zambom et al., 2019). These reciprocal effects can help explain the lasting activation of these systems, as well as the perpetuation of renal fibrosis in GT-treated rats. An additional possibility is represented by the transformation of endothelial cells and/or macrophages into myofibroblasts, a process observed in experimental models of fibrosis, such as adriamycin nephropathy, unilateral ureteral obstruction, and, most pertinent to our findings, AKI to CKD transition (Li et al., 2007; Tang et al., 2020).

In summary, rats that received GT for 9 days showed evident signs of AKI, with tubular necrosis, loss of renal function, and intense renal inflammation. Although structural and functional changes had regressed 30 days later, some important elements of inflammation persisted, along with activation of the RAS, the NF-κB system, and the NLRP3 pathway. One hundred and eighty days after the administration of GT, albuminuria, glomerular injury, and interstitial fibrosis had recurred, accompanied by evidence of sustained activation of the RAS, the NF-κB system, and the NLRP3/IL-1β pathway. These observations indicate that long-term renal fibrosis does occur after gentamicin-induced renal injury, and may be mediated, and perpetuated, by continuing renal activation of the RAS and of innate immunity. Targeting the NF-κB and/or the NLRP3 inflammasome systems may represent a new strategy in the effort to prevent the progression of renal fibrosis and, in particular, the AKI-CKD transition.

## Data Availability Statement

The original contributions presented in the study are included in the article/supplementary material, further inquiries can be directed to the corresponding author.

## Ethics Statement

The animal study was reviewed and approved by the Research Ethics Committee of the Faculty of Medicine of University of São Paulo.

## Author Contributions

AA, FZ, OF-N, KO, VA, SA, AS, DM, CF, and RZ carried out the experiments and analyzed the data. RZ, CF, and NC conceived the research project. All authors contributed to the article and approved the submitted version.

### Conflict of Interest

The authors declare that the research was conducted in the absence of any commercial or financial relationships that could be construed as a potential conflict of interest.
